# Nonlinear restructuring of patterned thin films by residual stress engineering into out-of-plane wavy-shaped electrostatic microactuators for high-performance radio-frequency switches

**DOI:** 10.1038/s41378-023-00549-5

**Published:** 2023-06-07

**Authors:** Rayan Bajwa, Heba Saleh, Milad Shojaeian, Ibrahim Tekin, Murat Kaya Yapici

**Affiliations:** 1 Faculty of Engineering and Natural Sciences, Sabanci University, 34956 Istanbul, Turkey; 2 Sabanci University SUNUM Nanotechnology Research Center, 34956 Istanbul, Turkey; 3 Department of Electrical Engineering, University of Washington, 98195 Seattle, WA USA

**Keywords:** Electrical and electronic engineering, Other nanotechnology

## Abstract

Electrostatic microelectromechanical (MEMS) switches are the basic building blocks for various radio-frequency (RF) transceivers. However, conventional cantilever-based designs of MEMS switches require a large actuation voltage, exhibit limited RF performance, and suffer from many performance tradeoffs due to their flat geometries restricted in two dimensions (2D). Here, by leveraging the residual stress in thin films, we report a novel development of three-dimensional (3D) wavy microstructures, which offer the potential to serve as high-performance RF switches. Relying on standard IC-compatible metallic materials, we devise a simple fabrication process to repeatedly manufacture out-of-plane wavy beams with controllable bending profiles and yields reaching 100%. We then demonstrate the utility of such metallic wavy beams as RF switches achieving both extremely low actuation voltage and improved RF performance owing to their unique geometry, which is tunable in three dimensions and exceeds the capabilities of current state-of-the-art flat-cantilever switches with 2D-restricted topology. As such, the wavy cantilever switch presented in this work actuates at voltages as low as 24 V while simultaneously exhibiting RF isolation and insertion loss of 20 dB and 0.75 dB, respectively, for frequencies up to 40 GHz. Wavy switch designs with 3D geometries break through the design limits set by traditional flat cantilevers and provide an additional degree of freedom or control knob in the switch design process, which could enable further optimization of switching networks used in current 5G and upcoming 6G communication scenarios.

## Introduction

High-performance switching elements are crucial to realize efficient wireless communication networks, where they are extensively used to route radio-frequency (RF) signals via transmission lines^1–4^. Conventionally, solid-state devices (e.g., transistors and diodes) are utilized as RF switches; however, they suffer from high DC power consumption and RF losses^3^. Meanwhile, with the current high-data rate 5G communication standards, the allowable margins for losses originating from the switches are becoming increasingly narrow. In such scenarios, the traditional solid-state switches can no longer meet emerging performance requirements^1^.

Therefore, in an attempt to achieve ideal RF switching, many solutions have been proposed, including microelectromechanical system (MEMS) electrostatic switches and 2D material-based atomic-scale switches^5–18^. Atomic-scale memristive switches show excellent potential for RF applications due to their high bandwidth, low power consumption, and fast response^19^. However, the power handling capability of memristive switches is limited, and their development is still in its infancy, with many bottlenecks in the way of wafer-scale production needing to be resolved^19,20^.

Alternatively, MEMS-based passive switching technology is quite mature, offering high linearity and extreme RF performance^3^. Among the various available design topologies of MEMS switches, fixed-free cantilever switches offer simple design, compact size, and fast switching speed^1^. Nevertheless, the voltages required to actuate such microrelays are generally large compared to solid-state switches^3^. In addition, due to a basic cantilever-based structure, a gap between the suspended switch beam and the underlying fixed actuation electrode and RF line defines both RF transmission and the required actuation voltage, which results in an inherent tradeoff between the actuation voltage and RF performance. To elaborate further, although a reduced gap directly reduces the required actuation voltage, it simultaneously degrades the RF performance by reducing the RF isolation in the up-state (i.e., the unactuated state). To minimize this fundamental tradeoff, a straightforward approach is to structurally modify and optimize the switch beam for a low spring constant, which results in lower actuation voltage but also lowers the beam strength and affects the reliability^1^. Correspondingly, many strategies exploiting various electrical configurations have been reported to overcome intrinsic switch performance tradeoffs, including series-shunt configuration and a series combination of multiple switches^3,11^. However, they all come at the expense of either increased design complexity or increased insertion losses (IL)^1,3^.

**Fig. 1:**
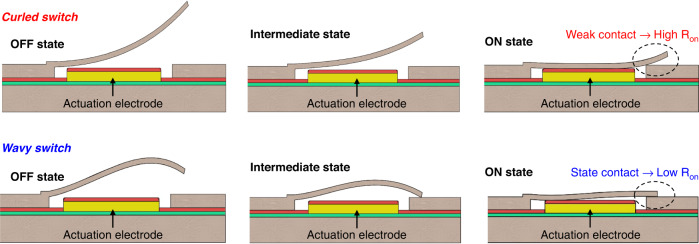
Wavy switch versus curled switch. Schematic diagram elaborating the operation of the proposed wavy switches and other curled 3D cantilever switches available in the literature

Another promising approach for improving switch performance is to convert traditional in-plane switch geometries into out-of-plane three-dimensional (3D) switches, which could allow us to break through the performance limitations set forth by existing flat-cantilever designs^6,7^. In one related study, bent-up cantilever switch beams with a low gap near the anchor point were reported to overcome the tradeoff between RF isolation and actuation voltage^6^. Although making the gap near the anchor point small and placing the DC electrode near the anchor might help actuate the switches at low voltage in a “zipping” motion^21^, the free end of such cantilever switches is prone to having weaker contact with the underlying RF line due to its curled-up topology (Fig. 1). This consequently increases the on-state resistance. To alleviate this problem, additional electrostatic forces are generally required that fully flatten the switch beam and tighten its contact with the RF line^7^. Hence, when used in actual circuits, these switches typically require voltage overdrive and thus demonstrate a large “effective” pull-in voltage. This phenomenon has been well demonstrated in another work, where a bent-up switch was fabricated with the aim of improving RF isolation, which exhibited electromechanical actuation at 35 V. However, it was observed that a voltage overdrive of up to 80 V was required to fully flatten the bent-up free end of the beam and enable sufficient contact with the RF output line^7^. Therefore, despite the potential of stress-induced bending technology that allows maneuvering the switch geometry in three dimensions to maximize switch performance in ways not possible with 2D flat-cantilever switches, existing 3D cantilever switches were unable to fully utilize the concept as they mostly restrict their bending to only one direction (upward), thus limiting the overall degree of freedom.

Here, attempting to fully harness the potential of the intrinsic stress-induced bending concept in designing 3D cantilever switches, we report a development route for a new class of MEMS switches, which we term “wavy switch”, by controllably leveraging the intrinsic stresses in the metallic structural layers of the cantilever and achieving out-of-plane bending in multiple directions (upward and downward). The switches utilize a combination of chromium and copper metals, which are widely employed in the IC industry, to realize for the first time, cantilevers with predictable wavy profiles through a simple high-yield fabrication process. We show that by using such wavy beams as RF switches, the conflicting relationship between RF isolation and actuation voltage can be effectively eliminated (which is not achievable with existing flat-cantilever switches). In addition, these wavy actuators also achieve stable contact with the underlying RF line upon actuation and are free from any voltage overdrive requirements, overcoming limits of existing 3D cantilever switches^7^ (Fig. 1). Furthermore, compared with state-of-the-art MEMS switches for mm-wave applications, we experimentally validated that our switches demonstrate excellent RF isolation and offer significantly lower actuation voltages.

## Results and discussion

### Controllable fabrication of wavy cantilevers

**Fig. 2:**
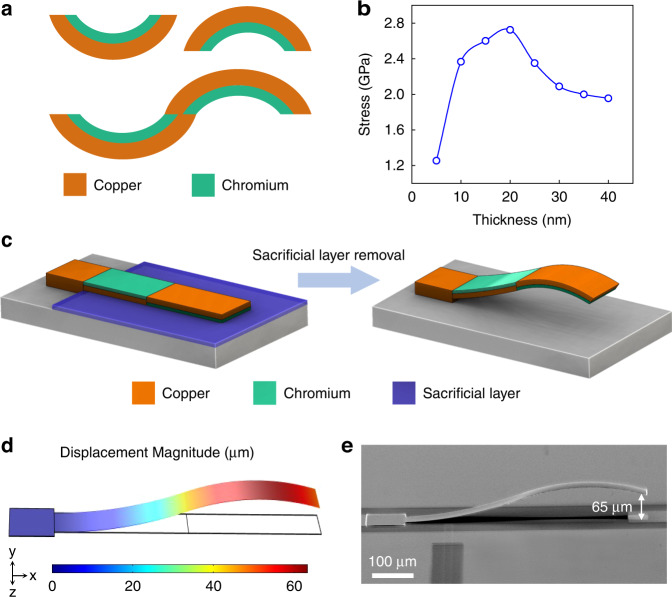
Structural design of wavy cantilevers. **a** Stress-induced bending in Cu and Cr thin-film stacks. **b** Characterized variation of residual stress in evaporated Cr with film thickness (as a convention, tensile nature of stress in indicated by positive stress values). **c** Assembly of flat cantilever into a wavy beam through a combination of Cu and Cr thin films. **d**–**e** Simulated bending profile and SEM image of a 600-µm-long wavy cantilever achieved by modulating the stress in Cu-Cr thin-film stack

Metallic thin films, when deposited on a substrate, often develop residual stresses that depend on the type of deposition technique as well as the deposition parameters^22^. By stacking multiple thin films with different residual stresses, a stress gradient can be created along the thickness, through which out-of-plane bending can be induced in thin films upon their release from the substrate. Relying on this concept, various bending profiles can be achieved using evaporated copper (Cu) and chromium (Cr) thin films by carefully managing their stress values and thicknesses (Fig. 2a). This occurs because these materials naturally exhibit a large stress gradient, i.e., the tensile stress in Cr is larger than that in Cu^23^.

Accordingly, we used evaporated copper and chromium thin films to fabricate wavy cantilevers. In addition to providing a large stress mismatch, Cu and Cr films are also highly compatible with standard IC fabrication, thus allowing for monolithic integration. To achieve controllable bending, we first characterized the stresses in Cr and Cu films based on Stoney’s equation (detailed in Supplementary Information Section S1). Evaporated chromium has been shown to possess a large tensile stress at low thicknesses (below 50 nm), while the tensile stress in Cu is generally low over a broad range of thicknesses^22,23^. Consistent with the literature^23^, we observed a large stress variation in the Cr thin film with the thickness (Fig. 2b), whereas the stress in the Cu film remained stable between 60 and 80 MPa for thicknesses up to 1 µm.

Considering its low stress and high conductivity suitable for electronic applications, Cu was utilized as the main structural layer of the cantilever beam, while high-stress Cr nanolayers were deposited atop and below the Cu layer to modulate the bending along the beam length and achieve a wavy profile. In particular, the thicknesses of the Cu and Cr films were adjusted to induce an upward bending moment in the first half of the cantilever beam close to its fixed end while inducing a downward bending moment in the other half near the free end, thereby allowing the assembly of the initially flat cantilever (adhered to the substrate via a sacrificial layer) into a wavy-shaped beam, as shown in Fig. 2c. A cross-sectional view of the proposed wavy cantilever is shown in Supplementary Fig. S2, in which the thicknesses of the Cu layer, bottom Cr layer and top Cr layer are labeled *t*_*Cu*_, *t*_*Cr_bottom*_ and *t*_*Cr_top*_, respectively. In addition, to accurately predict the bending profiles of the cantilevers, we established a finite element method (FEM) simulation setup based on quasistatic structural modeling^24^ (detailed in Supplementary Information Section S3). The bending simulation result and SEM image of a 600 µm long beam fabricated using the proposed strategy are provided in Fig. 2d, e, where a clear waviness in the beam shape can be observed with a tip deflection (TD) of ~65 µm.

**Fig. 3:**
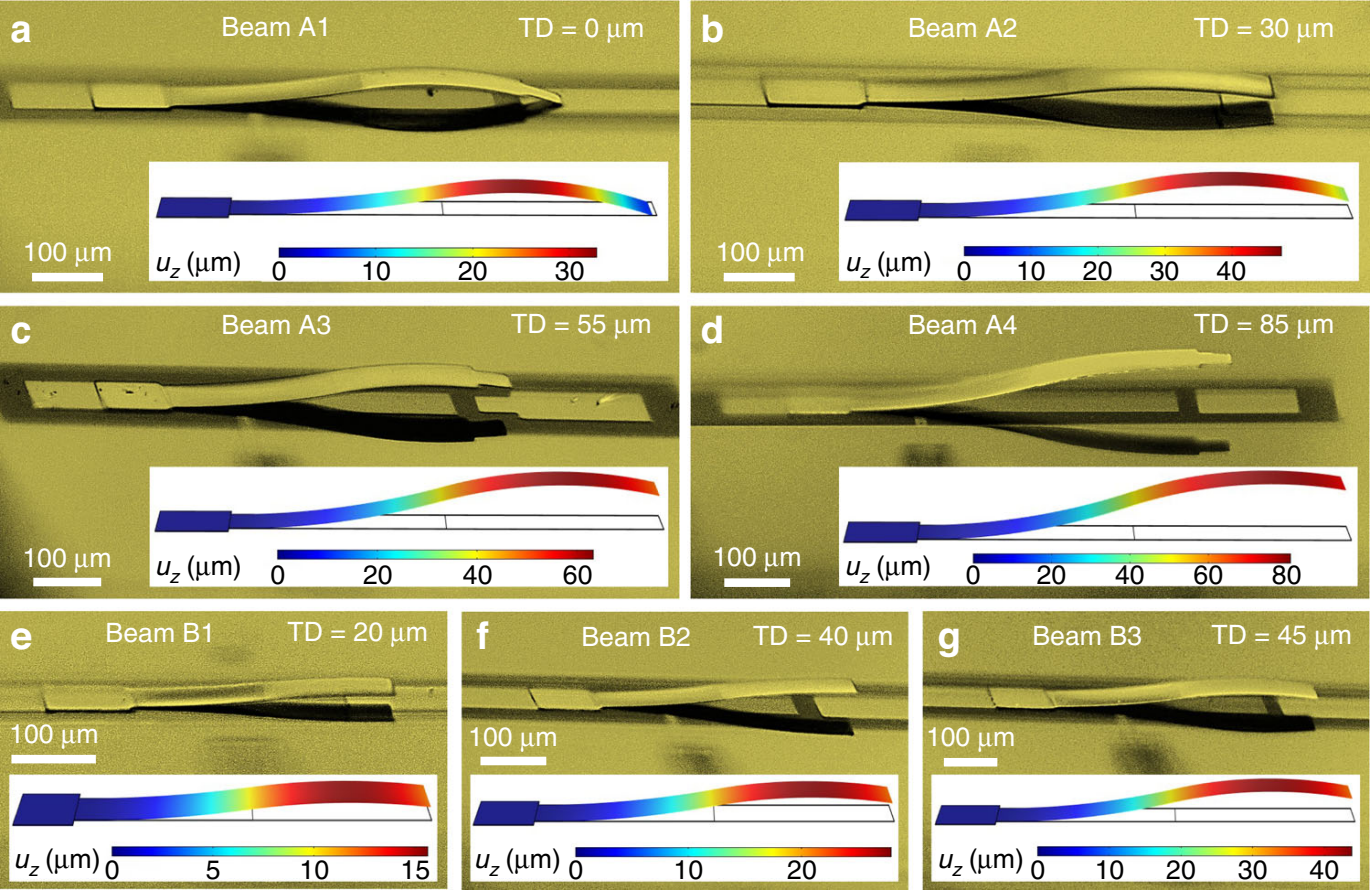
Bending controllability in wavy structures. Simulated and measured variations in wavy beam shape with **a**–**d** different stress conditions; and **e**–**g** different beam lengths

To effectively utilize stress-induced bending, ensuring controllability over bending profiles is very critical. To achieve this, we designed cantilevers with varying geometries, which allowed us to comprehensively validate the accuracy of simulations and controllability of fabrication. In total, seven types of beams were designed (A1-A4 and B1-B3), among which A-type beams have the same length and width but different Cu and Cr layer thicknesses (*t*_*Cu*_, *t*_*Cr_bottom*_ and *t*_*Cr_top*_) and thus different stress conditions. In contrast, the B-type beams possess the same stress conditions; however, they differ in length. The geometrical parameters of all 7 beam designs are listed in Supplementary Table S1. The corresponding fabrication and simulation results are reported in Fig. 3. As shown in Fig. 3a–d, a large variation in the overall wavy profile is achieved by slightly adjusting the thicknesses of the top and bottom Cr nanolayers (exact layer thicknesses are provided in Supplementary Table S1). Specifically, 600 µm long A-type cantilever beams with varying stress conditions exhibited tip deflections ranging from 0 to 85 µm. Furthermore, the fabrication and simulation results for B-type beams are given in Fig. 3e–g, which illustrate a significant influence of beam length on the bending profile. This length-based curvature tuning is of particular importance since it provides an easy way to tailor the bending profile, as opposed to modifying the thicknesses and corresponding residual stresses, which always brings up a certain degree of uncertainty due to the inevitable nonuniformity of the fabrication process and equipment. These well-matched simulation and fabrication results verify the accuracy of the simulation setup and demonstrate a controllable platform for the fabrication of wavy structures, which allowed us to precisely control the profile of wavy switches reported in subsequent sections.

### Wavy cantilevers as high-performance RF switches

**Fig. 4:**
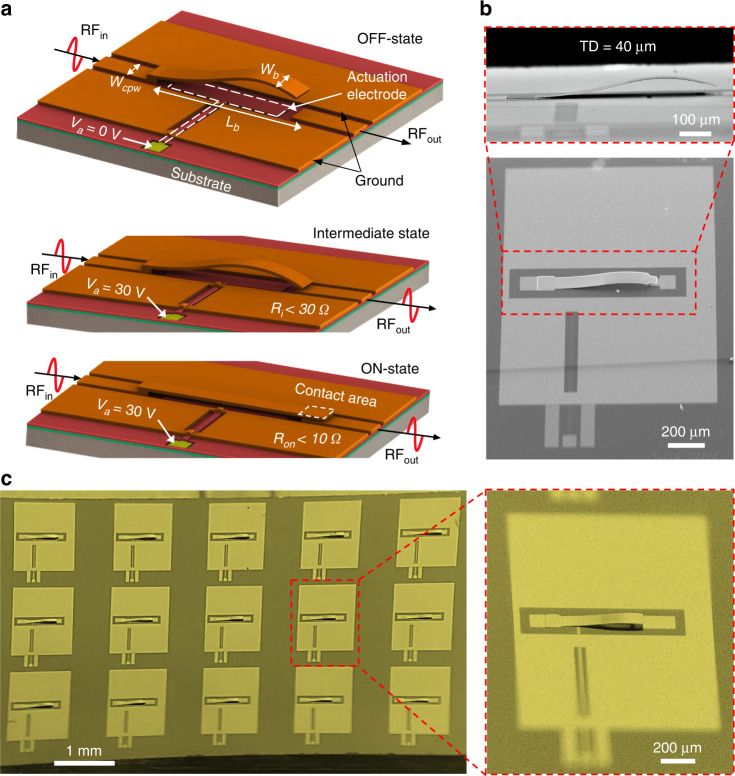
Wavy switch design and implementation. **a** Design and operating principle of the proposed wavy switches. **b** SEM image of the proposed wavy switch with a TD of 40 µm. **c** A sample with a high fabrication yield containing 15 fully functional RF switches

The structural design and ON/OFF operation of the proposed wavy RF switches are depicted in Fig. 4a. The wavy cantilevers were embedded in a coplanar waveguide (CPW) configuration to realize in-line series RF switches, and *L*_*b*_, *W*_*b*_ and *W*_*cpw*_ represent the length of the switch, width of the switch, and width of the CPW signal line, respectively. Notably, both upward and downward curvatures in the wavy switch beam play important roles. The upward bending elevates the beam and provides a large air gap from the RF output line, resulting in high OFF-state RF isolation. At the same time, the gap between the switch beam and underlying actuation electrode is small near the anchor point, which in turn triggers electrostatic pull-in at lower values of actuation voltage (*V*_*a*_). Although pull-in can be triggered at a low voltage in such a configuration, a large additional voltage beyond pull-in is required to fully flatten the bent-up distal end of the beam and enable stable contact with the underlying RF line^7^. To address this issue, we induced a downward curvature at the distal end of the beam to ensure sufficient contact with the RF output line without the need for any voltage overdrive. We verified this behavior via experiments where our proposed switches resulted in very low on-state resistances (*R*_*on*_ < 10 Ω) at the pull-in point. Meanwhile, no significant improvement in the resistance was observed when the actuation voltage was increased up to double the original pull-in voltage (*V*_*pi*_), as shown in Supplementary Fig. S4. This essentially validates the existence of a stable contact at the pull-in point, which is achieved by downward bending in the beam. Furthermore, the proposed wavy switch establishes contact with the RF output line prior to fully snapping to the underlying actuation electrode (Supplementary Videos S2 and S3) due to a downward curvature at its free end (as shown in the “intermediate state” of Fig. 4a), which further illustrates the natural ability of wavy switches to achieve strong contact with the underlying output line upon actuation. With this principle of operation, the wavy beams reported herein overcome the inherent performance tradeoff between RF isolation and actuation voltage by offering their simultaneous optimization.

Relying on the above concept, we designed a total of eight wavy switches (X1-X4 and Y1-Y4) with varying lengths and widths to thoroughly investigate the effectiveness of the wavy switch design. However, the beam thickness and corresponding stress conditions were kept constant, with the aim of obtaining tip deflections above 30 µm in all designs to achieve sufficient RF isolation. Details on the structural parameters of all 8 wavy switch designs are provided in Supplementary Table S2. Following their design, wavy switches were fabricated using a simple high-yield fabrication process. Figure 4b shows an SEM image of the fabricated wavy switch with a length, width and TD of 600 µm, 80 µm and 40 µm, respectively. Moreover, a diced sample containing 15 fully functional wavy switches is shown in Fig. 4c, demonstrating 100% fabrication yield.

### Fabrication of wavy switches

**Fig. 5:**
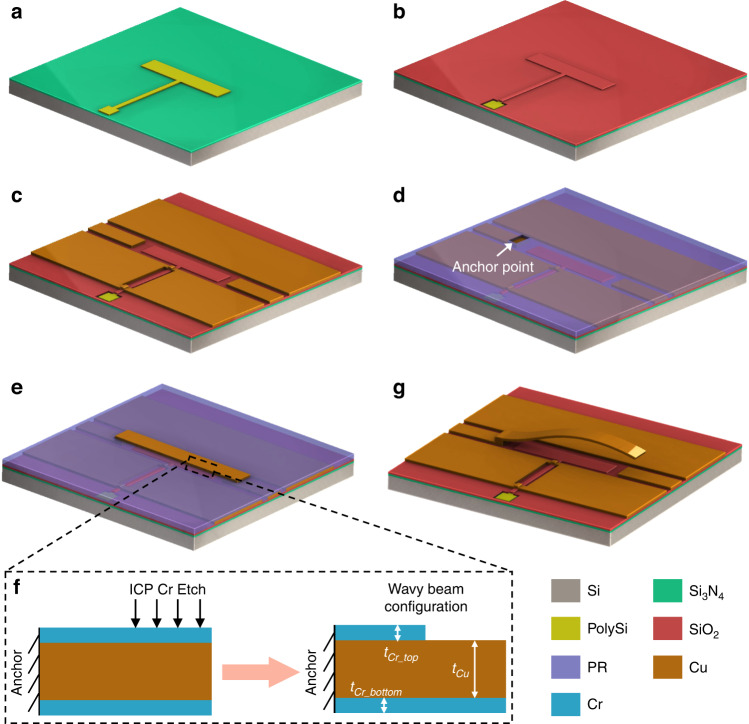
Fabrication of wavy RF switches. **a** Coating HR-Si substrate with Si_3_N_4_ passivation layer and pattering of polysilicon DC actuation electrode. **b** SiO_2_ insulation layer for DC actuation electrode. **c** Pattering CPW structure using copper layer. **d** Photoresist sacrificial layer patterning. **e** Pattering of thin-film stack made of Cu and Cr layers to define the beam. **f** Side-view of wavy beam prior to its release shows patterning of top Cr layer using ICP etching, to modulate the stress gradient along beam length. **g** Sacrificial layer removal, yielding a wavy-shaped switch beam

A stepwise fabrication process for the proposed wavy switches is shown in Fig. 5. Compared with the fabrication of conventional MEMS switches, our fabrication requires only one additional lithography step (photoresist patterning and etching) to achieve complex and controllable wavy profiles. Briefly, we used high-resistivity silicon (HR-Si) as a substrate to minimize the RF losses. A doped polysilicon (PolySi) layer was used to define the DC actuation electrode (Fig. 5a), which was then insulated with a SiO_2_ layer (Fig. 5b). Subsequently, a copper layer was deposited and patterned to specify the ground and signal lines of CPW (Fig. 5c), followed by the pattering of the photoresist (PR) sacrificial layer to define the anchor points (Fig. 5d). Next, a thin-film stack containing three layers (Cu layer, bottom Cr layer, and top Cr layer) was deposited and patterned to specify the beam shape (Fig. 5e). At this stage, the top Cr layer of the beam was patterned again to modulate the stress conditions along the beam length, as shown in Fig. 5f. It is worthwhile to note that top Cr layer pattering is the only additional step required to convert traditional flat-cantilever switches into wavy-shaped switches. In addition, the proposed fabrication scheme does not require any special processing or materials; instead, we utilize common Cu and Cr metals along with typical surface micromachining techniques to achieve a wavy profile, and therefore, the proposed fabrication process is fully IC compatible. Finally, the beam was released by etching away the underlying sacrificial layer, which resulted in a wavy cantilever (Fig. 5g). The remaining details regarding the fabrication are provided in “Materials and methods”.

### Electromechanical response of wavy switches

**Fig. 6:**
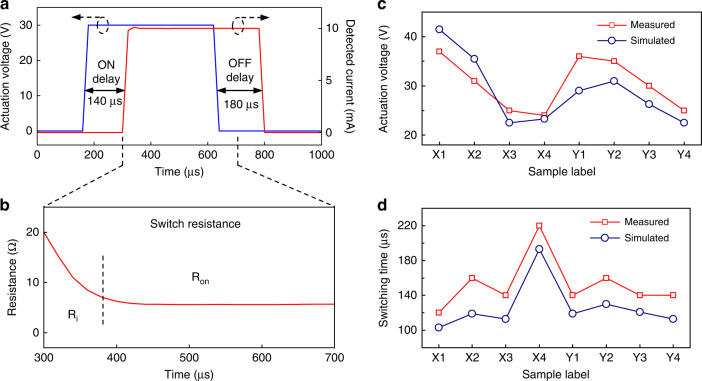
Electromechanical performance of wavy switch. **a** Characterized ON-OFF operation of the fabricated X3-type wavy switch. **b** Switch resistance during the switching ON period. **c** Measured and simulated actuation voltages of all 8 types of fabricated switches. **d** Measured and simulated switching times of all 8 types of fabricated switches

Figure 6a displays the characterized ON-OFF operation of the fabricated X3-type wavy switch, indicating turn-on and turn-off times of 140 µs and 180 µs, respectively. The switching time measurements were performed at actuation voltages slightly higher than the actual pull-in voltages of the switches. In addition, Fig. 6b shows the evolution of switch resistance during switching, where first a relatively high resistance value was observed (~20 Ω), which then converged to a stable lower value (below 10 Ω) after a few tens of microseconds. This observation validates the existence of an ‘intermediate state’ in which only the tip of the switch contacts the output line prior to full flattening of the switch beam. It should be noted that the measured switch resistance includes the resistance of the metallic switch beam (<0.1 Ω), probe resistance (2–3 Ω), and switch contact resistance.

Fabricated wavy switches with varying geometries were investigated via both simulations and experiments to systematically analyze their electromechanical response (details on the simulation setup are given in Supplementary Information Section S3). Supplementary Videos S1 and S2 show the characterized and simulated switching operation of the proposed wavy switches. In addition, the measured and simulated switching times and actuation voltages for all types of fabricated switches are provided in Fig. 6c, d, which shows that the actuation voltage is limited well below 40 V for all designs and reaches the minimum value (23 V) for switch type X4. Progressing from switch type X1 to X4, the beam length increases from 300 µm to 600 µm while the other geometrical parameters remain constant. Despite the large tip deflections of beams with larger lengths (Supplementary Table S2), which could severely weaken the impact of the electrostatic actuation force, an increase in the beam length still lowered the required actuation voltage (Fig. 6c), through the utility of the proposed wavy-shaped designs, i.e., the actuation voltage decreased from 37 to 23 V as the length increased from 300 to 600 µm. However, an increased switch length resulted in a slower switching speed (Fig. 6d) since a longer switch beam inherently exhibits a lower mechanical resonance frequency, which increases the switching time^4^. In particular, the switching time increased from 120 to 220 µs as the length increased from 300 to 600 µm. On the other hand, the lengths of all B-type designs are the same, and thus, they exhibit similar switching times of ~140 µs (Fig. 6d). As seen from Supplementary Table S2, the designed B-type switches differ in their widths, and varying width results in a varying overlapping area between the switch beam and underlying actuation electrode. Therefore, as the beam width increased from 50 to 80 µm, the corresponding actuation voltage decreased from 36 to 25 V due to the increased overlapping area.

### High-frequency performance of wavy switches

**Fig. 7:**
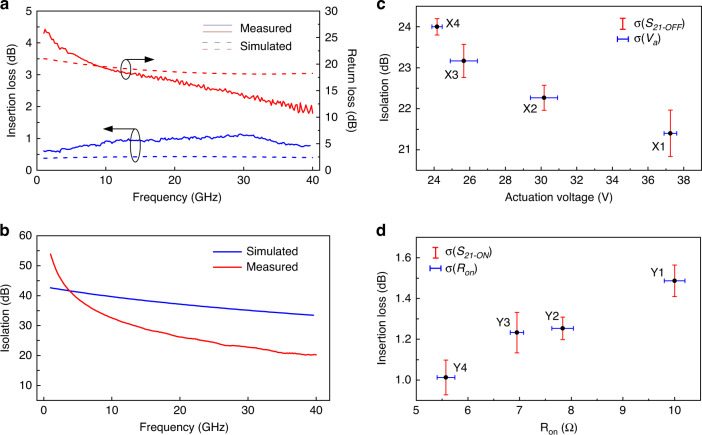
High-frequency performance of wavy switch. **a** Measured and simulated insertion loss and return loss of X3-type switch. **b** Measured and simulated RF isolation of X3-type switch. **c** Variation of actuation voltage (*V*_*a*_) and RF isolation (*S*_*21-OFF*_ @30 GHz) among X1-X4-type switches. **d** Variation of ON-state switch resistance and insertion losses (@30 GHz) among Y1-Y4-type switches. Standard deviations are calculated by measuring each switch design three times from three different samples

Simulated and measured high-frequency scattering parameters (S-parameters) of the X3-type fabricated switch in the ON and OFF states are shown in Fig. 7a, b, respectively. These results show that the switch exhibits very high isolation (*S*_*21-OFF*_ > 20 dB), with an insertion loss (*S*_*21-ON*_) of ~1 dB and a return loss (*S*_*11-ON*_) better than 10 dB for frequencies up to 40 GHz. Similar trends were also observed in the measured and simulated RF characteristics of the rest of the fabricated switches, as shown in Supplementary Fig. S8. It is worthwhile to note that there is a certain discrepancy between the measured and simulated results, which we attribute to nonidealities in material properties, fabrication conditions, and measurement environment. In addition, the switch contact was also considered ideal in simulations, while during actual operation, losses at the contact region contribute greatly to the overall switch insertion loss and are highly sensitive to ambient factors such as humidity; thus, unavoidable mismatches were observed^3,25^. Next, we characterized the linearity of the proposed switches by analyzing their two-tone third-order intermodulation distortion (IMD3) at 2.4 GHz, which revealed the input third-order intercept point (IIP3) beyond 48 dBm (see “Materials and methods” and Supplementary Figs. S9 and S10).

To better analyze the effectiveness of wavy designs, we studied the variation trends of RF isolation (at 30 GHz) and actuation voltage among various fabricated switches with varying lengths (Fig. 7c). As seen from the figure, by simply increasing the length of wavy switches (from switch type X1 to X4), simultaneous optimization of RF isolation and actuation voltage is achieved, which is nearly impossible with conventional flat-cantilever switch designs. To verify the measurement repeatability, we measured multiple devices with the same geometry and quantified the performance variation using the standard deviation (σ, indicated by error bars in Fig. 7c). We observed the variation in actuation voltages of identical switches to be less than 5%, while that in isolation values was below 1 dB (Fig. 7c). These variations may be attributed to the variability in the measurement environment and processing conditions during device fabrication.

Another key parameter for RF-MEMS switches is the insertion loss (IL) in the ON state, which primarily depends on the contact resistance of the switch^4^. A key factor affecting the contact resistance is the contact area, where a larger contact area yields a lower contact resistance^4^. Relying on this principle, we varied the beam width among our Y-type samples, which resulted in varying contact areas (from 50 µm × 50 µm to 50 µm × 80 µm for switches Y1-Y4). We observed an improvement in contact resistance, i.e., *R*_*on*_ minus probe resistance (2 Ω), and corresponding insertion loss with increasing contact area (Fig. 7d), where contact resistance reached the minimum (~3 Ω) for the Y4-type switch with an associated insertion loss of ~1 dB at 30 GHz, owing to its largest contact area. Moreover, to verify the repeatability of the measurements, we measured the inconsistency between the results obtained from the same type of switches, as shown by the σ error bars drawn in Fig. 7d, which indicate a performance variation of less than 5%. It should also be noted that since insertion losses depend heavily on the contact region and its resistance, the effect of wavy topology on insertion losses is insignificant.

### Reliability and power handling of wavy switches

**Fig. 8:**
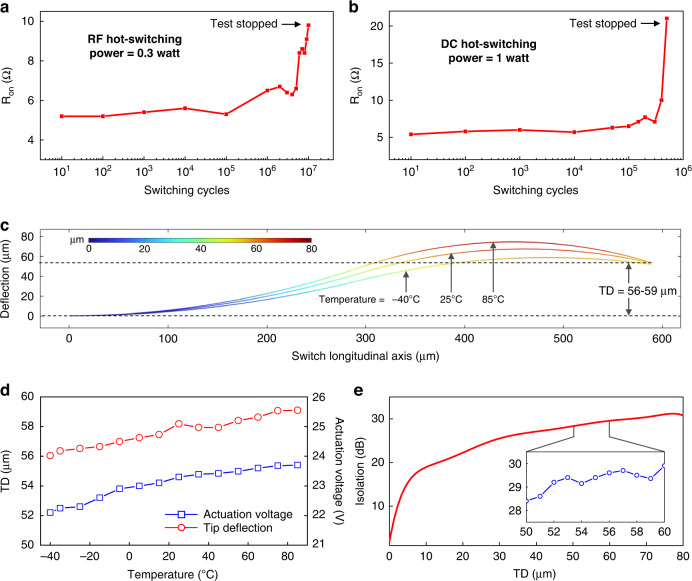
Reliability of the proposed wavy switches. Lifetime characterization **a** under an RF power of 0.3 W; **b** under a DC power of 1 W. Tests were stopped when the ON-state resistance (*R*_*on*_) crossed 10 Ω. **c** Sensitivity of the wavy profile of switch X4 to temperature (under the industrial temperature range, i.e., −40 °C to 85 °C). **d** Variation in tip deflection (TD) of switch X4 for temperatures ranging from −40 to 85 °C. **e** Simulated RF isolation of switch X4 at different degrees of tip deflection at a frequency of 30 GHz

The reliability of RF-MEMS switches is often a concern due to their mechanical nature, which causes wear at the contact region under long-term usage for various reasons, such as microwelding, material transfer, and contact hardening^3,26^. Therefore, assessing the reliability and power handling ability of the proposed wavy actuators is crucial to demonstrate their feasibility for practical applications. First, we characterized the lifetime of our switches under hot-switching conditions (i.e., keeping the power on while turning the switches on and off), where we used switch X3 for experiments. We hot-switched the actuator with 0.3 W of RF power (frequency = 2.4 GHz) for 10 million cycles (in alignment with typical test durations^11,12,14,27,28^) at a switching frequency of 2 kHz and simultaneously measured its on-state resistance at different intervals (hot-switching experiment is illustrated in Supplementary Video S4). These switch cycling results are provided in Fig. 8a and show that the switch resistance remained stable (between 5 and 7 Ω) for up to 4 million cycles, after which it increased to approximately 10 Ω at 10 million cycles. The test was stopped at 10 million cycles, when the resistance reached the failure criteria (*R*_on_ > 10 Ω).

To further test the switch’s robustness to hot-switching conditions, we cycled our X3 switch at higher power (1 W of DC power). These results are given in Fig. 8b. In principle, DC power degrades the switch contact faster than RF power, primarily because it causes a larger amount of material transfer at the contact region^29^. As shown in Fig. 8b, our switch cycled with a stable ON-state resistance for up to 0.3 million cycles. After this point, the resistance started increasing sharply and ultimately exceeded the failure criteria (*R*_on_ > 10 Ω) after 0.5 million cycles, which is similar to that of the other reported switches with acclaimed high reliability under DC hot-switching conditions^30^.

It should be noted that all the testing is performed with unpackaged switches in an open-air environment, and the switch lifetime can be improved by adding a hermetic seal^31^. Considering that contact degradation is the most typical cause of switch failure, the use of harder materials (e.g., Ru) at the contact region can also improve the switch lifetime^32^, as opposed to softer materials e.g., Au or Cu (used in this work), which are commonly preferred due to their lower resistivity. Furthermore, the use of multiple switch contacts that result in a “zipping” actuation has also been reported in the literature^30^ to simultaneously achieve high reliability and low contact resistance.

The power handling capability of RF-MEMS switches is yet another figure-of-merit that is interrelated with the switch lifetime and reliability. Two major criteria that limit the power handling of RF-MEMS switches are self-actuation and RF latching failures^33^. Self-actuation refers to unwanted actuation of the switch under large RF power, which creates a large potential difference between the hanging switch beam and underlying output RF line. Self-actuation primarily depends on the overlap area and the gap between the switch beam and RF line and hence is typically more pronounced in membrane-type shunt switches than in series switches^4^. The proposed wavy beams are series switches with a small overlap area with the underlying RF line. In addition, owing to the out-of-plane geometry, the gap between the RF line and wavy switch beams is also relatively large (>20 µm) compared to conventional flat-cantilever series switches, which indicates the built-in immunity of the proposed switches to self-actuation. To validate this, we applied up to 70 V DC between the switch beam and the RF line; however, no detectable deformation was observed in the switch geometry. Notably, 70 V peak-to-peak voltage translates to approximately 40 dBm or 10 W of RF power in a 50 Ω system, which is quite high. The other factor that limits the power handling of MEMS switches is unintentional RF latching or stiction, which occurs after application of high RF power even though the DC actuation voltage is removed. However, this failure is observed more in capacitive switches that have a dielectric layer at the contact between the RF line and switch beam, which creates an electrostatic potential difference holding the switch in contact^4^. In our testing with power up to 1 W, no undesired latching issues were observed.

We also studied the sensitivity of the proposed switches to temperature since multilayer wavy cantilevers can exhibit a significant temperature-induced strain due to the thermal expansion coefficient mismatch between different layers (Cu and Cr) in a thin-film stack that constitutes the wavy shape. A deformed wavy shape under thermal stress can essentially modify the RF isolation and actuation voltage of the switch. Therefore, to verify the resilience of the wavy switch performance against thermal stresses, we analyzed the variations in RF isolation and actuation voltage over the industrial temperature range, i.e., −40 °C to 85 °C. For analysis, we considered the X4-type switch because this device has the greatest length and hence will exhibit the maximum strain among all switch designs (subject to the same thermal conditions). We established a thermomechanical simulation environment (described in Supplementary Information Section S3), where we varied the temperature and recorded the corresponding changes in the wavy profile (Fig. 8c). We observed that an increase in temperature slightly increases the degree of waviness and vice versa for the case of decreasing temperature; however, the variation in tip deflection (TD) is negligible. In particular, TD increased from 56 µm to 59 µm as the temperature increased from −40 to 85 °C (Fig. 8d). For deformed wavy profiles, we then extracted the actuation voltage using electromechanical simulations (detailed in Supplementary Information Section S3), which reveal that the required actuation voltage varies less than 5% from its value at room temperature (25 °C) to its value at −40 °C and at 85 °C (Fig. 8d).

Fundamentally, the RF isolation of the switch is mainly defined by the overlap area between the switch beam and the underlying RF line. Therefore, to understand the influence of temperature on the RF isolation of switches, we first simulated the isolation (at 30 GHz) of switch X4 under different amounts of tip deflection. As seen from the results given in Fig. 8e, RF isolation degrades sharply with TD only when TD is below 10 µm; however, in all our wavy switches, TD is maintained above 20 µm. This in turn illustrates that the variation in the TD of the proposed switches due to thermomechanical deformation will not have a significant effect on RF isolation. For instance, considering switch X4 with TD varying from 56 to 59 µm over the temperature range −40 to 85 °C, the corresponding change in RF isolation is less than 1.5 dB (Fig. 8e, inset). Based on these results, we conclude that although wavy switches can be more prone to unwanted thermally induced strains, their performance remains virtually invariable over a wide range of temperatures.

### Comparison with the state-of-the-art

To evaluate the performance improvement, we compared two of our best-performing switches (X3 and X4) with the existing state-of-the-art for in-line series MEMS switches operating at mm-wave frequencies, as shown in Table 1. All compared designs require larger actuation voltages (>50 V) than the proposed wavy switches, which operate at only 24 V. At the same time, our wavy switches also exhibit a relatively large RF isolation (at 40 GHz) when compared to the other designs, thus offering simultaneous optimization of isolation and actuation voltage that cannot be guaranteed with existing flat-cantilever type geometries. To further highlight the effectiveness of wavy switches in optimizing the isolation-voltage tradeoff, we benchmarked our switches against the existing designs through a new performance index parameter (*k*) that we formulated, which incorporates both RF isolation (*S*_*21-OFF*_) and actuation voltage (*V*_*a*_) into a simple ratio of |*S*_*21-OFF*_ | /*V*_*a*_. This means that better or higher isolation (a larger absolute value of *S*_*21-OFF*_) and lower actuation voltage (a smaller value for the DC pull-in voltage) yield a larger *k* parameter. In view of the current 5G communication scenario, we calculated the performance index (*k*) for all compared designs at 40 GHz, as reported in Table 1. Due to the unique wavy geometry, the proposed switches outperform all the other compared designs in terms of the performance index (*k*) by a large margin. In addition, as seen in Table 1, the insertion loss of wavy switches is in a reasonable range; however, it is not the lowest among the compared designs. This is because the insertion losses primarily depend on contact resistance, which is slightly higher in our switches since we employ a Cr/Cu bilayer contact material, as opposed to conventional gold contacts that inherently provide a lower contact resistance due to their better conductivity and low hardness. Therefore, by simply adding the gold contacts to our wavy designs, the lowest achievable insertion losses can certainly be reached. The only drawback of wavy topology is the large switching time compared to existing designs, which can be attributed to its relatively long length.

**Table 1:** Performance comparison of the proposed switches with existing works

Ref.	Actuation voltage	Isolation (dB)		IL (dB)		Switching Time	Hot-switched lifetime	Performance index (*k*)	
		20 GHz	40 GHz	20 GHz	40 GHz		(millions—MM)	20 GHz	40 GHz
^8^	50 V	24	15	0.7	1	–	–	0.48	0.3
^14^	80 V	20	14	0.6	0.6	10.6 µs	100 MM @ 0.1 W	0.25	0.18
^11^	50 V	50	36	0.35	0.43	30.4 µs	100 MM @ 1 W	1	0.72
^7^	60 V	18	14	0.3	0.7	3 µs	–	0.3	0.23
^18^	60 V	25	17.5	0.9	0.9	2.2 µs	–	0.41	0.29
^34^	80 V	30	24	0.25	0.4	–	–	0.37	0.3
^12^	60 V	10	–	1.1	–	103 µs	3 MM @ 0.1 W	0.16	–
^27^	89 V	20	–	1	–	10 µs	100 MM @ 0.3 W*	0.22	–
^28^	80 V	21	–	0.9	–	200 µs	4 MM @ 30 mW*	0.26	–
^5^	15 V	21	–	0.8	–	1.8 µs	725 MM @ 1 mW	1.4	–
Switch X3	25 V	27	20.4	0.9	0.75	140 µs	10 MM @ 0.3 W	1.08	0.82
Switch X4	24 V	29	20.2	0.7	0.94	220 µs	–	1.20	0.84

^*^Device is hermetically packaged

## Conclusion

In conclusion, we have introduced a new class of radio-frequency MEMS switches based on wavy-shaped microactuators fabricated through a simple and fully IC-compatible process. We characterized our devices in detail, including their electromechanical and high-frequency responses. Our in-line series wavy switches actuate at voltages as low as 24 V while maintaining large RF isolation and low insertion loss for frequencies up to 40 GHz, demonstrating a significant improvement over current state-of-the-art devices and indicating a massive potential for application in emerging high-data-rate communication systems. Through the application of a wavy bending profile in originally flat-cantilever switches, a strict fundamental performance tradeoff between RF isolation and actuation voltage can be successfully omitted, thus liberating an additional degree of freedom in the design process. Furthermore, we embedded our wavy microactuators within the simplest topology of MEMS switches (in-line series configuration) to better demonstrate the proposed concept, while our switches can be easily integrated into any other switch configuration to achieve even further improvement in performance. In the future, this capability may be shown by integrating wavy switches in shunt configuration, a topology that is known to provide better RF performance at higher frequencies.

## Materials and methods

### Wavy switch fabrication

We first deposited a 1-µm-thick silicon nitride (Si_3_N_4_) layer on a 500-µm-thick high-resistivity silicon substrate for passivation. To define the actuation electrode and pad, a polysilicon layer was then sputtered and lithographically patterned (etched using SF_6_ gas). Next, to electrically insulate the actuation electrode, we deposited a 400-nm-thick silicon dioxide layer and patterned it using a commercial etchant (buffered oxide etchant) to uncover the underlying actuation pads. We then used the lift-off technique along with thermal evaporation to pattern a thin-film stack containing a 300-nm copper layer with a 5-nm chromium adhesion layer to define the CPW structures. Afterward, the photoresist (AZ5214E) was spin-coated as the sacrificial layer with a thickness of 500 nm (photoresist was diluted with an appropriate solvent to achieve such a thickness). The patterned photoresist layer was then hardened at 170 °C on a hotplate for 45 min to improve its chemical resistance to other materials used in the subsequent processes (e.g., acetone). We then deposited and patterned the switch beam layer containing Cu and Cr thin films (layer thicknesses vary as listed in Supplementary Table S2) using the thermal evaporation and lift-off technique. Next, the top Cr layer was patterned using photolithography and reactive ion etching (with Cl_2_ gas) to modulate its thickness and corresponding stress conditions along the beam length. Finally, we removed the hard-baked photoresist layer in a photoresist remover at 80 °C to release the wavy switch beams. The released samples were rinsed thoroughly first in water and then in isopropyl alcohol (IPA) and dried at room temperature to avoid any stiction issues.

### Electromechanical characterization

The experimental characterization was performed using a Keysight source measurement unit (SMU) with two channels, as shown in Supplementary Fig. S5. The actuation voltage was provided to the switch through channel 1, while with channel 2, a voltage (100 mV) was applied to two terminals of the signal line with the switch connected in series between them. Upon actuation under the action of applied voltage from channel 1, the current passing through the switch was sensed from channel 2 (current was limited to 10 mA), from which both transient and steady-state characteristics of the switch were extracted.

### High-frequency characterization

The radio-frequency response of the switches was analyzed with a 0–50 GHz vector network analyzer, where the two-port S-parameters of switches in both the ON and OFF states were recorded. The switches were designed to have a ground-signal-ground (GSG) configuration at both ports, and thus, GSG RF probes were used for measurements (Supplementary Fig. S6). Prior to measurements, GSG probes were calibrated using the short-open-load-thru method (SOLT) with a commercial calibration substrate. During high-frequency measurements, a DC actuation signal was also applied to switches using the SMU to control the switch actuation, and to avoid interference from any unwanted DC signal, DC blocks were used at both ports. Last, the power from the VNA was varied from −5 to 10 dBm in the experiments, but no obvious change in the results was observed since MEMS switches inherently have high linearity.

### Lifetime and linearity experiments

The measurement setup for characterizing the switch lifetime and linearity is shown in Supplementary Fig. S7, which consists of two signal generators, a coupler, GSG/DC probes, a 20 dB attenuator, a two-channel SMU, and a spectrum analyzer. To perform IMD3 tests for analyzing device linearity, we actuated the switch using DC voltage supplied by SMU (channel 1) and DC probes, supplied two RF signals with power 13 dBm from two signal generators to the switch via a coupler, and recorded the output spectrum with the spectrum analyzer. The output power was attenuated by 20 dB with an attenuator to ensure that the spectrum analyzer operated in the linear region, and the total loss from input to output was 23 dB. Moreover, the RF signals were set 20 MHz apart with a center frequency of 2.4 GHz. As shown in Supplementary Fig. S9, no third-order harmonic in the output was observed at 13 dBm of input power, at which the difference between the output peaks and base signal level was 70 dB. Since increasing the input power further beyond 13 dBm was not possible due to equipment limits, we considered the output signal base level (−80 dBm, as shown in Supplementary Fig. S9) as the third-order component amplitude. With this assumption, we then estimated OIP3 and IIP3, as illustrated by the third-order intercept chart given in Supplementary Fig. S10.

For lifetime measurements under RF hot-switching conditions, we used a signal generator with GSG probes to supply 25 dBm (~0.3 W) RF power to switches and SMU (channel 1) for generating an actuation signal at a cycling frequency of 2 kHz. To measure switch resistance during testing, we occasionally turned off the RF source and used the SMU (channel 2) for resistance readings. For DC hot-switching experiments, we removed the RF source and GSG probes and supplied DC power to switch from the SMU (channel 2), while the rest of the measurement setup remained the same as that of the RF hot-switching tests.

## Supplementary information

Supplementary Information

Video of switch operation in action

Switch pull-in simulation

Variation of mesh during the simulated switching operation

Switch cycling operation, RF Hot Switching
