# Supplementary material for: Nonlinear restructuring of patterned thin films by residual stress engineering into out-of-plane wavy-shaped electrostatic microactuators for high-performance radio-frequency switches
Source: Microsyst Nanoeng. 2023 Jun 7;9:74. doi: 10.1038/s41378-023-00549-5 (PMC10247711; doi:10.1038/s41378-023-00549-5)
Supplement: Supplementary file 1 — Supplementary Information [file 41378_2023_549_MOESM1_ESM.docx]

**S1. Stress Characterization**

For residual stress measurements, we first measured the radius of curvature of bare silicon substrate using KLA-TENCOR P6 surface profiler. Next, we deposited the desired film thickness on the substrate and again measured its radius of curvature. Using these substrate radii of curvature before and after the thin film deposition, we estimated the residual stress in deposited thin film according to Stoney’s equation, expressed as:

|  | $\text{σ =}\frac{\text{E}\text{t}_{\text{1}}^{\text{2}}}{\text{6}\text{t}_{\text{2}}\text{(1-}\text{v}_{\text{s}}\text{)}}[\frac{\text{1}}{\text{R}_{\text{f}}}-\frac{\text{1}}{\text{R}_{\text{s}}}$] | (1) |
| --- | --- | --- |

where $\sigma$ is the amount of stress, *R_f_* is the radius of curvature after film deposition, *R_s_* is the radius of curvature of bare substrate, $t_{1}$ and $t_{2}$ are the thicknesses of substrate and film, *E* is the elastic modulus of the substrate, and $v_{s}$ is the Poisson’s ratio. Using this strategy, we characterized the residual stresses in evaporated copper and chromium films at thicknesses ranging up to 50 nm and 1 µm, respectively, as shown in Figure S1.


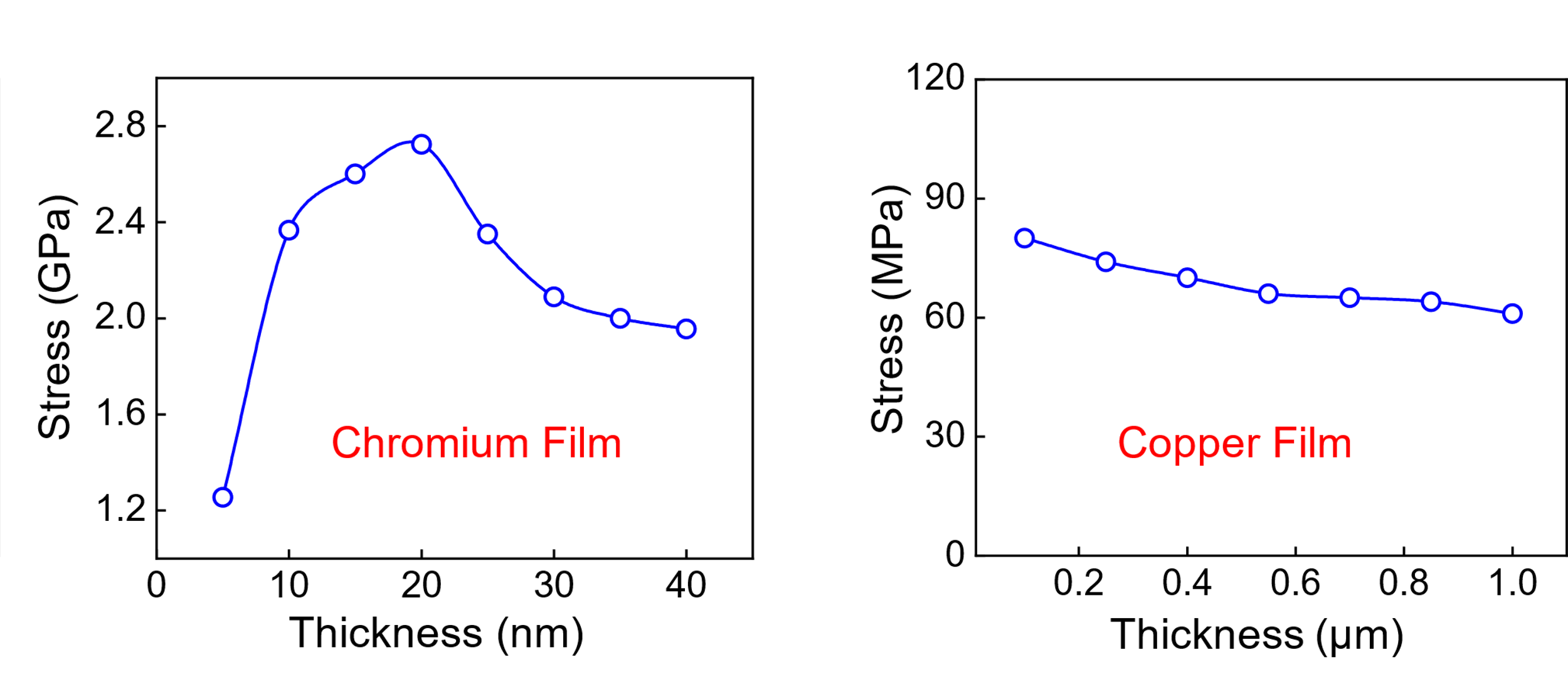


**Figure S1.** Characterized stresses in evaporated copper and chromium thin films.

**S2. Wavy Beam Configuration**

**
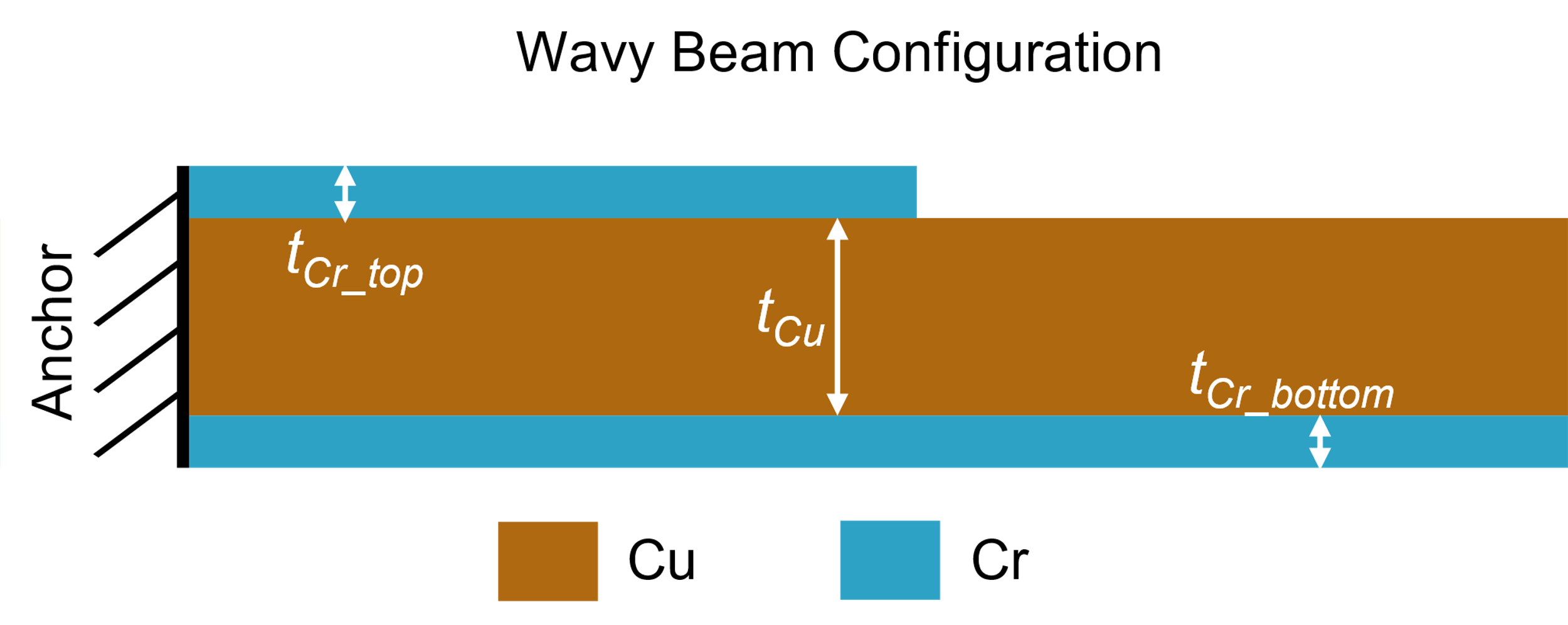
**

**Figure S2.** Cross-sectional view of wavy beam structure.

**S3. Finite Element Simulations**

***Bending Estimation***

To estimate the bending profiles, a simulation setup up was established in COMSOL Multiphysics 5.6 using its Structural Mechanics module. First, the 3D geometries were built with configuration shown in Figure S2, and relevant materials were assigned to the layers. The materials were assumed to be linearly elastic, however, the geometric nonlinearities were considered as the beams were expected to undergo a large deformation. Following the material assignment, a fixed boundary condition was assigned to anchor point, while the residual stresses measured with profiler (shown in Figure S1) were assigned to corresponding layers. To mimic the sacrificial release process, a moving boundary condition was assigned at the bottom edge of the beam, to deform the beam in a quasi-static manner^1^. For meshing, we use a 2D mesh at the top surface of the beam and then sweep it across the thickness to mesh to whole geometry. As recommended by COMSOL, such type of meshing reduces the computation time for beams with large aspect ratio, compared to the standard tetrahedral mesh. Finally, the stationary solver of COMSOL was used to compute the bending profiles.

***Electromechanical Simulation of Wavy Switches***

Electromechanical behavior of wavy switches was predicted using COMSOL Multiphysics 5.6 with physics type set to “Electromechanical”. Owing to its high computation cost, we performed the electromechanical simulation in 2D. Therefore, the above structural simulations were first repeated in a 2D environment to extract the 2D bending profiles of all switches listed in Table S2. In actual scenarios, stresses are isotropic and therefore the bending is uniform along both length and width of the beam. However, by restricting the simulations to 2 dimensions, we in fact ignored the bending effect along the beam width, since in beams where width is significantly smaller than the length, the bending profile is primarily defined by beam length^2^. Next, the deformed geometry was imported as the initial geometry in the electromechanical simulation setup where necessary materials and boundary conditions were assigned. The developed electromechanical simulation setup was dedicated to extract both stationary and transient responses of the switches under the applied actuation voltage (electrostatic force), which allowed us to obtain pull-in voltages and switching times, respectively. The pull-in study was performed using the stationary solver where applied voltage was increased in small increments (0.1 V) until the pull-in point was reached. On the other hand, the transient behavior was studied by using a time-dependent solver of COMSOL. To consider the fringing field effect, the gap between switch beam and actuation electrode was modeled as air with “moving mesh” condition. To model the contact of switch beam with insulated underlying actuation electrode, a combination of adhesion-repulsion (Lennard-Jones model of intermolecular forces) with the penetration penalty method for the beams was employed by defining the variables implemented as boundary load in solid mechanics physics^3^. The mesh type for the beam was set as “mapped” and that for the surrounding air was set as “triangular”. Since the gap between beam and underlying stationary electrode was large, the deformation of mesh elements in the gap increased dramatically as the beam moved towards the substrate. In such scenario, the software experienced an ill-posed condition labelled as “inverted mesh”. To address this problem, automatic remeshing was considered in the transient study, to maintain the mesh quality. Video S3 shows the variation of mesh during the simulated switching operation.

To ensure a reliable operation, it is crucial to study the stresses in switch beam during actuation. Therefore, in our simulations, we also recorded the maximum stress in switch beams during an actuation cycle, which generally occurs at the anchor point when the beam is fully actuated and flattened. In our switches, copper thin film acts as the main structural layer and for a reliable operation, the maximum stress in switches should be well-below the yield strength of copper. Fig. S4 shows the simulated variation of maximum stress with time in switch type X4, which exhibited the largest maximum stress during actuation among all switch types due to its large tip deflection. It can be seen from the figure that the maximum stress is contained below 130 MPa, which is significantly lower than the yield strength of copper i.e., around 400 MPa at thickness of 1 µm.


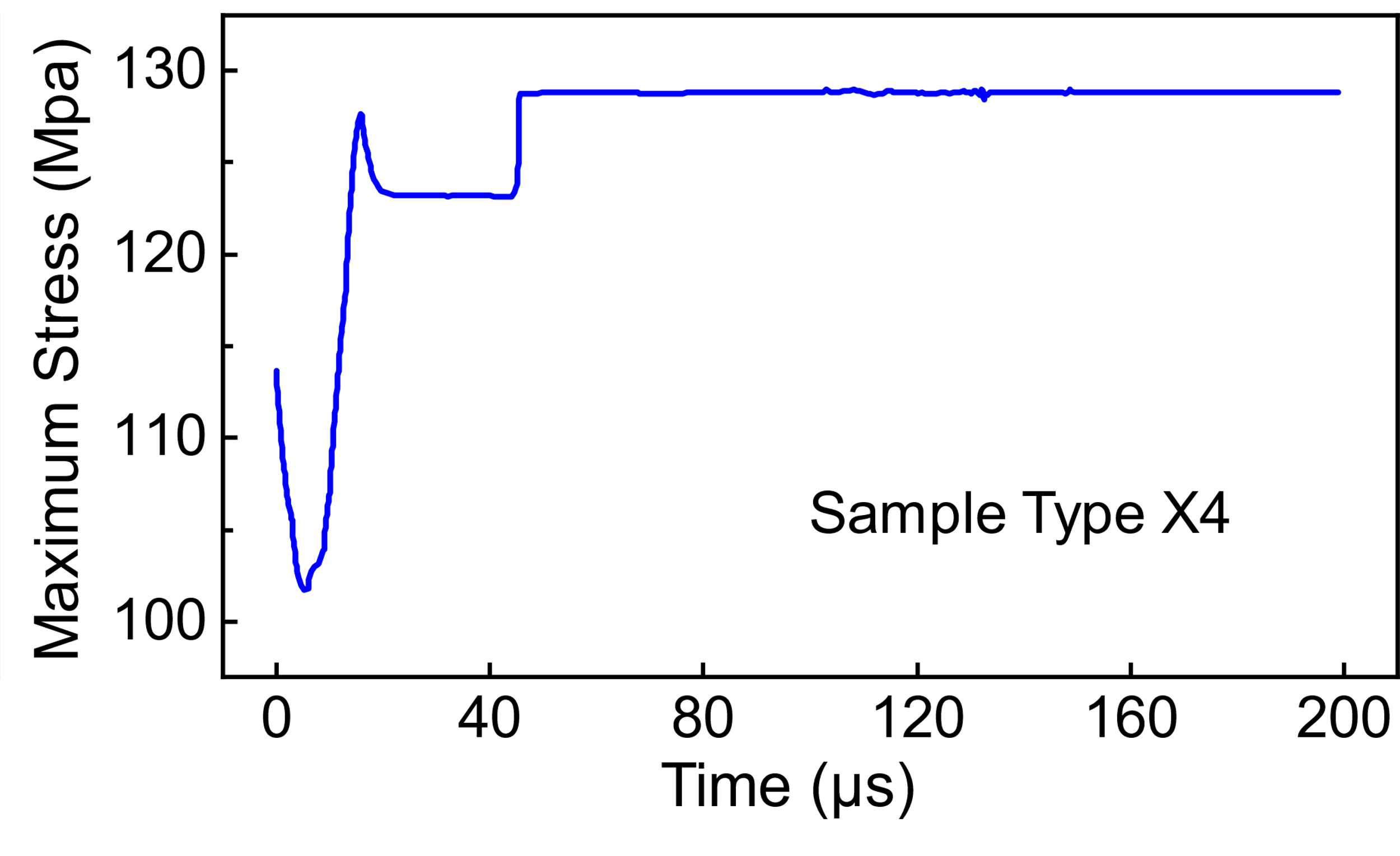


**Figure S3.** Maximum stress in Switch X4 during actuation.

***High-Frequency Simulations of Wavy Switches***

Following the electromechanical analysis, switch geometries in ON and OFF states were imported to HFSS for high-frequency analysis, where first the appropriate material properties were assigned. In addition, we enclosed the switch geometry in an air box which enables a consistent distribution of electromagnetic (EM) fields around the device to be simulated, thus artificially replicating the practical measurement environment. Moreover, we chose “Driven Modal” as the model type while the excitation type was selected as “Lumped Port”. Next, we configured a frequency sweep from 0-40 GHz, a range similar to that of our measurement system (vector network analyzer and RF probes). Lastly, the EM fields are solved by the built-in solver of HFSS relying on Maxwell’s equations over the specified frequency range, which yielded the final S-parameters of the switch.

***Thermomechanical Simulation***

To estimate the bending profile variation under thermal load, a simulation setup up was established in COMSOL Multiphysics 5.6 using its Structural Mechanics module. The bending profiles estimated with bending simulations (described above) were imported to software and materials were assigned to the layers. Then, we added thermal coefficient of expansion to Cu and Cr layers, which is necessary to simulate the thermomechanical behavior of beams. Moreover, the materials were assumed to be linearly elastic; however, the geometric nonlinearities were considered, as the beams were expected to undergo a large deformation under the thermal stress. After the material assignment, a fixed boundary condition was assigned to anchor point, and the temperature was introduced in the system as a boundary condition. Finally, tetrahedral meshing was used and the bending variation is simulated using the software’s stationary solver, where temperature was increased from -40°C to 85°C.

**S4. Parameters of Fabricated Wavy Test Structures and Switches**

**Table S1.** Geometrical parameters of various fabricated wavy beams.

| Beam Type | Length (µm) | Width (µm) | *t_Cr___top_* (nm) | *t_Cr___bottom_* (nm) | *t_Cu_* (nm) | Tip Deflection (µm) |
| --- | --- | --- | --- | --- | --- | --- |
| A1 | 600 | 80 | 13 | 10 | 850 | 0 |
| A2 | 600 | 80 | 17 | 13 | 850 | 30 |
| A3 | 600 | 80 | 19 | 13 | 850 | 55 |
| A4 | 600 | 80 | 21 | 13 | 850 | 85 |
| B1 | 300 | 70 | 19 | 13 | 850 | 20 |
| B2 | 400 | 70 | 19 | 13 | 850 | 40 |
| B3 | 500 | 70 | 19 | 13 | 850 | 45 |

**Table S2.** Parameters of fabricated 8 types of switch samples.

| Sample Label | *L_b_* (µm) | *W_b_* (µm) | *W_cpw_* (µm) | *t_Cr___top_* (nm) | *t_Cr___bottom_* (nm) | *t_Cu_* (nm) | Tip Deflection (µm) |
| --- | --- | --- | --- | --- | --- | --- | --- |
| X1 | 300 | 80 | 100 | 19 | 13 | 850 | 20 |
| X2 | 400 | 80 | 100 | 19 | 13 | 850 | 35 |
| X3 | 500 | 80 | 100 | 19 | 13 | 850 | 40 |
| X4 | 600 | 80 | 100 | 19 | 13 | 850 | 55 |
| Y1 | 500 | 50 | 100 | 19 | 13 | 850 | 40 |
| Y2 | 500 | 60 | 100 | 19 | 13 | 850 | 40 |
| Y3 | 500 | 70 | 100 | 19 | 13 | 850 | 45 |
| Y4 | 500 | 80 | 100 | 19 | 13 | 850 | 40 |

**S5. ON-state Switch Resistance at Various Voltage Levels**

**
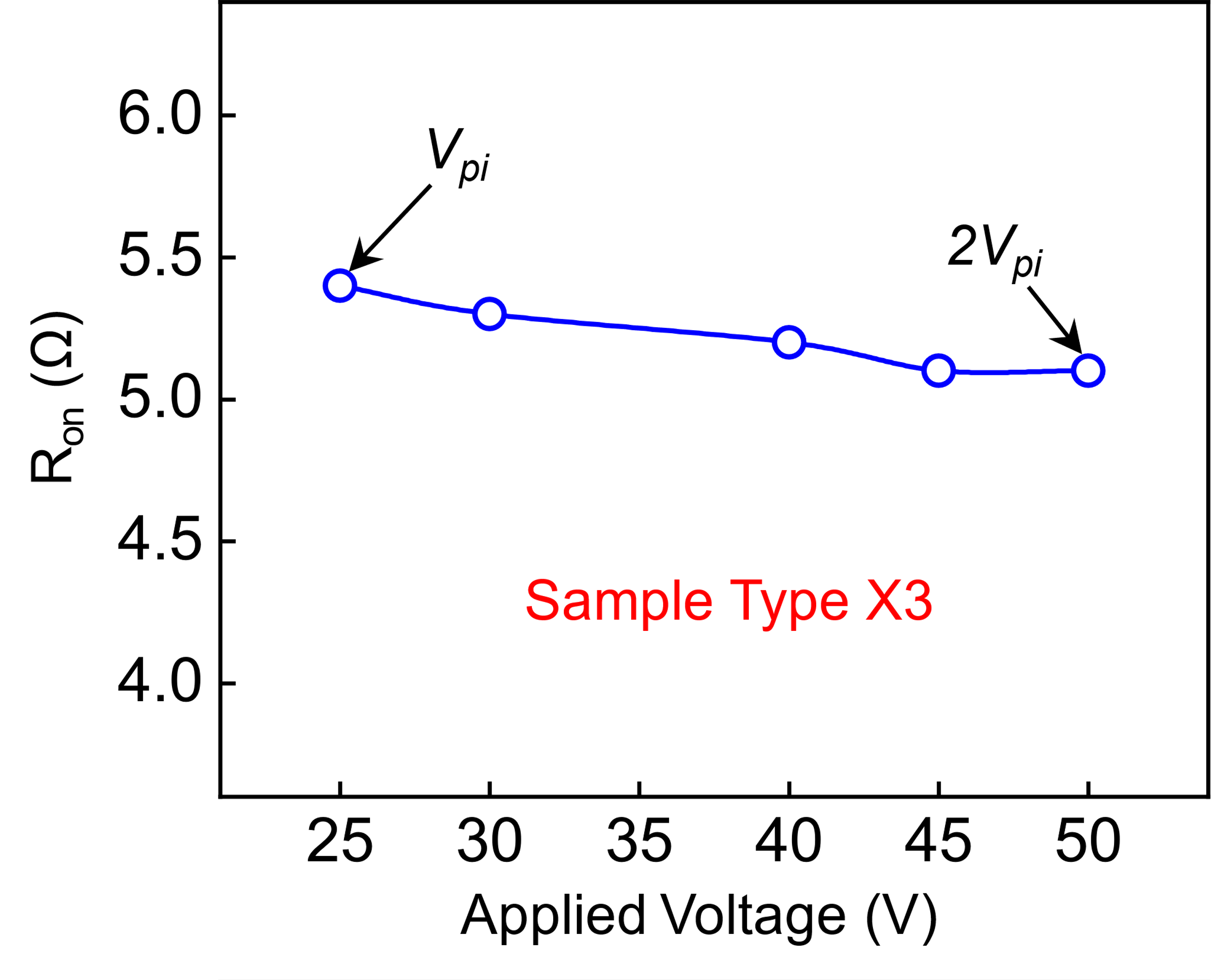
**

**Figure S4.** Variation of ON-state switch resistance with increasing actuation voltage.

**S6. Measurement Setup**


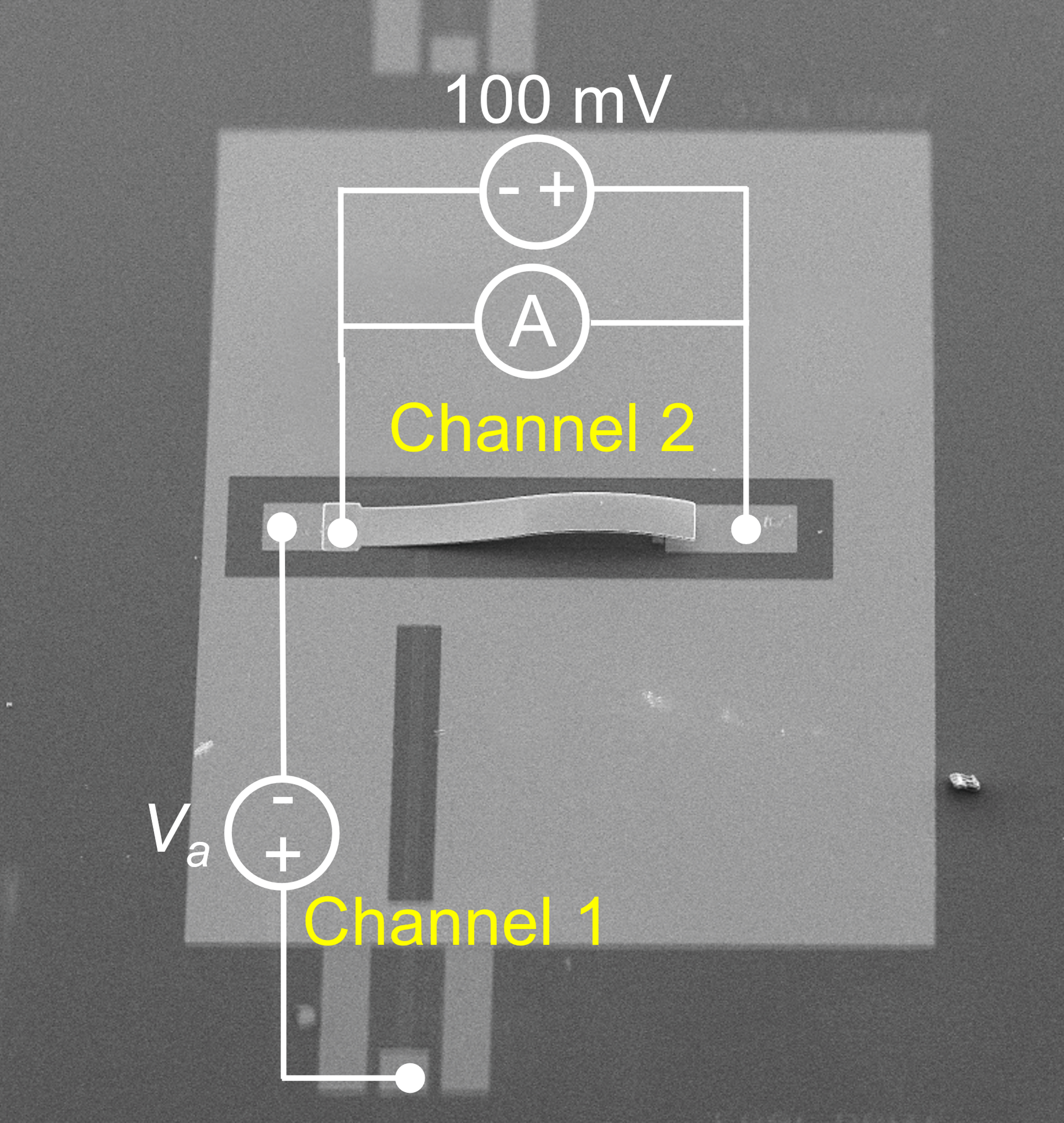


**Figure S5.** Electromechanical characterization setup using source measurement unit with 2 channels.


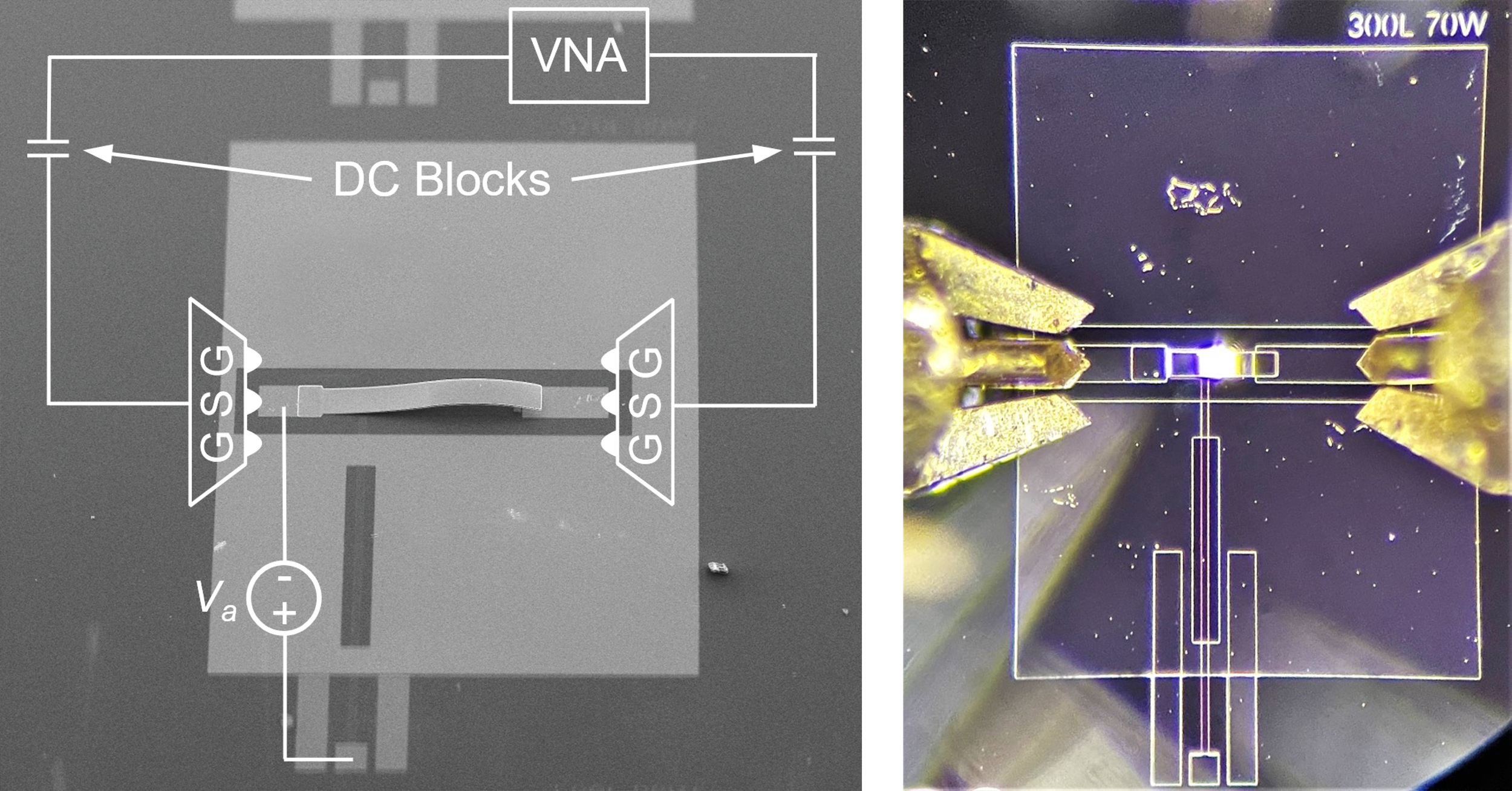


**Figure S6.** High-frequency characterization setup for the wavy switches.

**
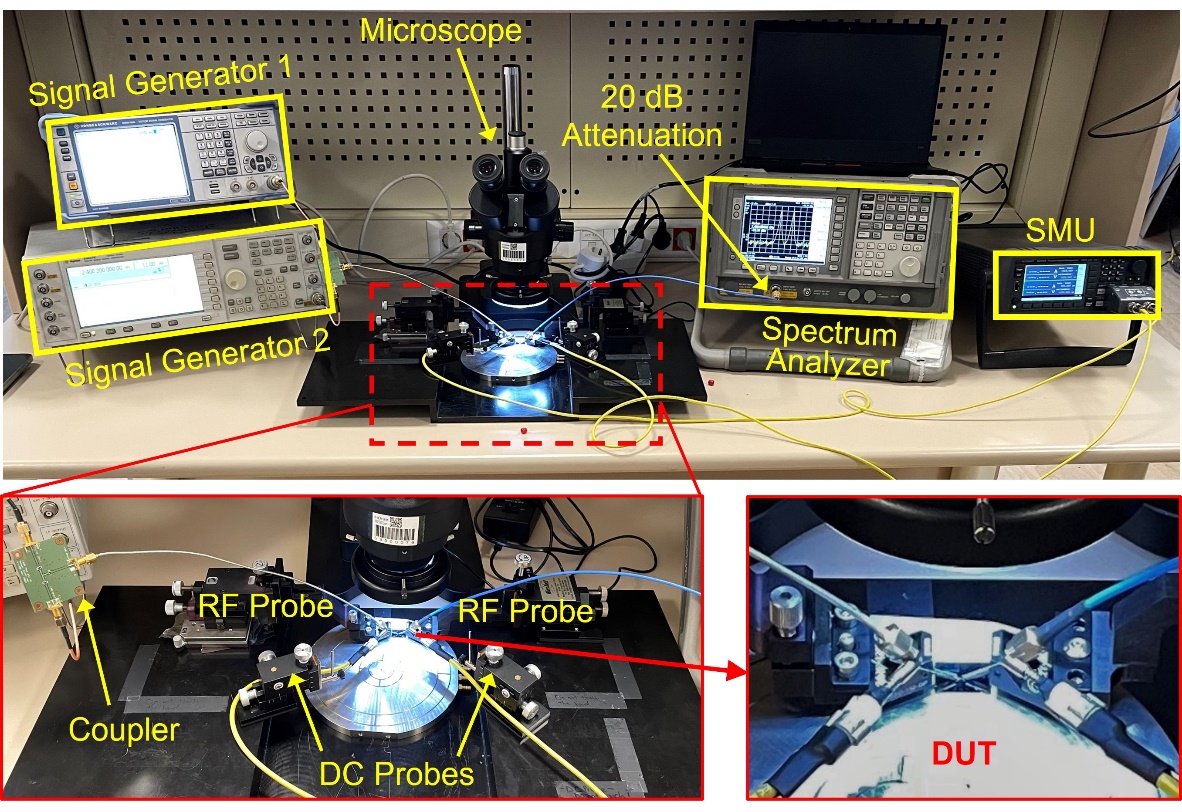
**

**Figure S7.** Experimental setup for characterization of switch lifetime and linearity.

**S7. Supplementary High-Frequency Measurement Results**

All the measured and simulated s-parameters for all 8 types of designs are plotted in Fig. S8. As can be seen from the figure, we also observed some discrepancies between the measured and simulated results. For instance, the insertion losses of Switch X3 and X4 are lower than that of Switch X1 and X2. However, since we embedded all of our switches in the transmission line of same length, the length of switch itself should have a minimal effect on the measured insertion loss (from one GSG probe to the other). That is, all X type switches X1-X4 should ideally have a very similar insertion loss as their contact area is the same, as can be verified from the measurement results. We would like to highlight that in series DC contact RF MEMS switches, contact resistance is the main factor controlling the insertion losses and it dominates the line losses^4^. This contact resistance is highly sensitive to environmental conditions, and without hermetic seal to switches, contact resistance can vary largely during measurements ^5,6^. That is to say, as we measured the switches unpackaged in open-air lab environment, although being minor, there are unavoidable/unpredictable differences in the measurement compared to simulations.

**
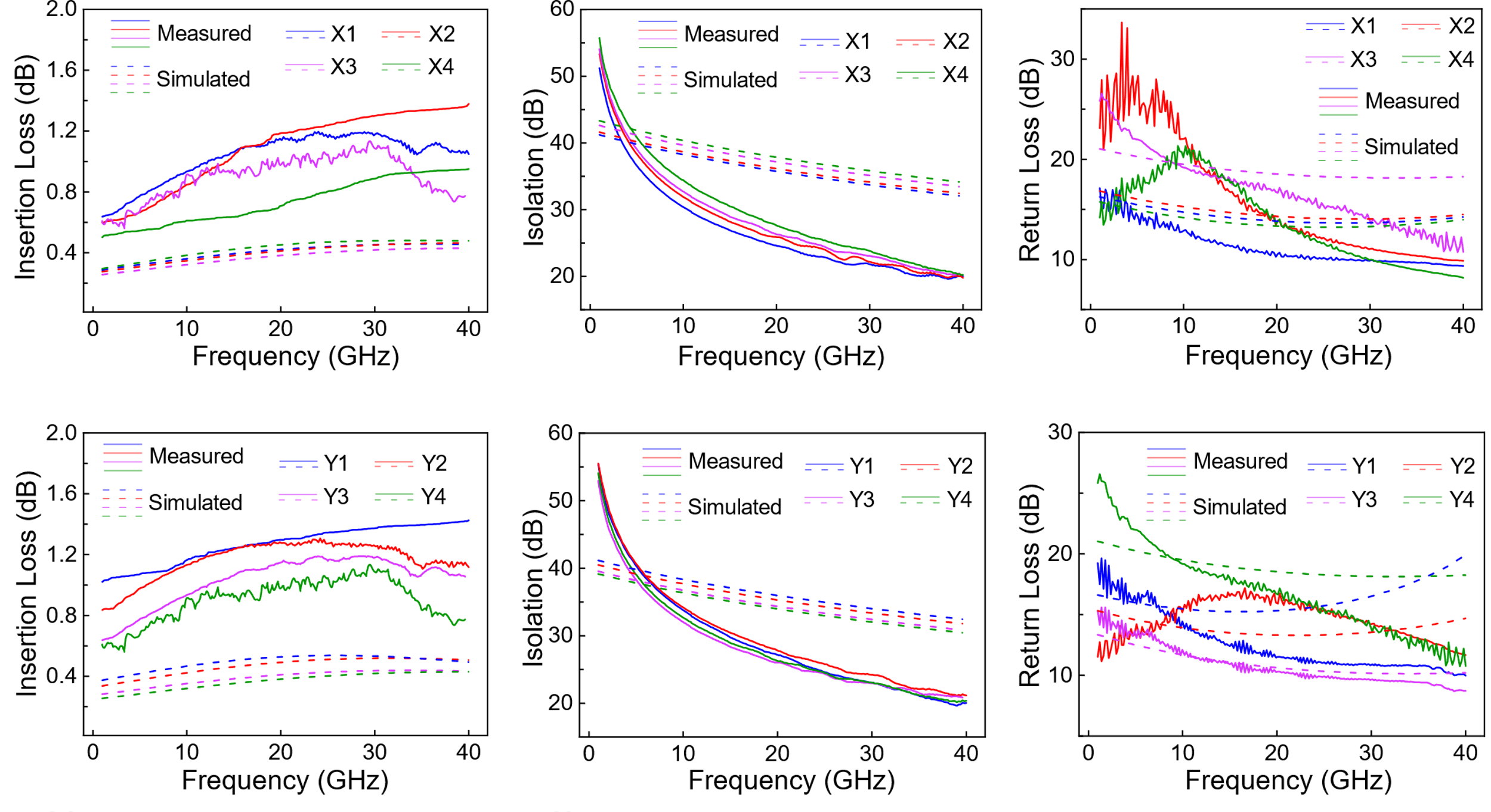
**

**Figure S8.** Measured high-frequency responses of all 8 types of fabricated wavy switches. Return loss corresponds to the on-state.


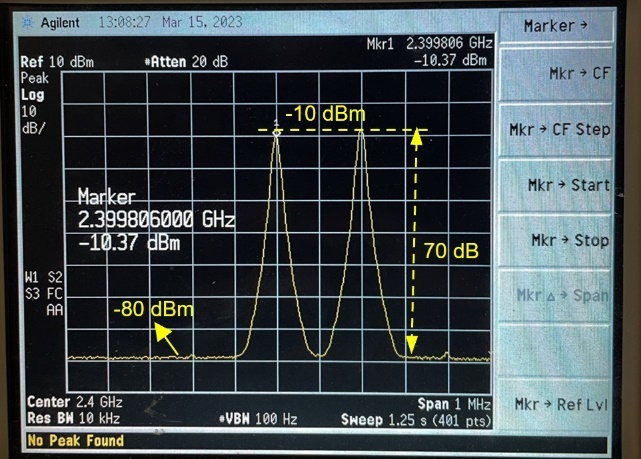


**Figure S9.** Output at spectrum analyzer during two tone IMD3 experiments for linearity characterization under an input power of 13 dBm.


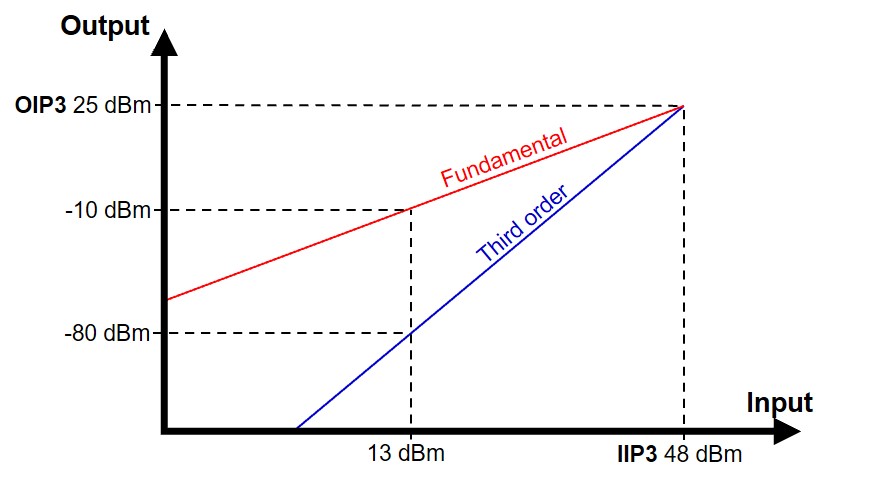


**Figure S10.** Characterized linearity of proposed wavy switches.

**References**

1 Huang, W., Koric, S., Yu, X., Hsia, K. J. & Li, X. Precision Structural Engineering of Self-Rolled-up 3D Nanomembranes Guided by Transient Quasi-Static FEM Modeling. *Nano Letters* **14**, 6293-6297, doi:10.1021/nl5026369 (2014).

2 Bajwa, R. & Yapici, M. K. Intrinsic stress-induced bending as a platform technology for controlled self-assembly of high-Q on-chip RF inductors. *Journal of Micromechanics and Microengineering* **29**, 064002, doi:10.1088/1361-6439/ab16bd (2019).

3 Sylves, K., Maute, K. & Dunn, M. L. Adhesive surface design using topology optimization. *Structural and Multidisciplinary Optimization* **38**, 455-468, doi:10.1007/s00158-008-0298-4 (2009).

4 Rebeiz, G. M. *RF MEMS: Theory, Design, and Technology*. (Wiley, 2003).

5 Qing, M. *et al.* in *Proc.SPIE.* 646305.

6 Rebeiz, G. M. & Muldavin, J. B. RF MEMS switches and switch circuits. *IEEE Microwave Magazine* **2**, 59-71, doi:10.1109/6668.969936 (2001).
